# Antitumour and Immunomodulatory Effects of Cu(II) Complexes of Thiobenzyhdrazide

**DOI:** 10.1155/MBD.2002.109

**Published:** 2002

**Authors:** Nand K. Singh, Saty B. Singh, Anuraag Shrivastav

**Affiliations:** 1 Department of Chemistry Banaras Hindu university Varanasi 221 005 India; 2 Department of Pathology & Saskatoon Cancer Centre College of Medicine University of Saskatchewan 20 Campus Drive Saskatoon SK S7N 4H4 Canada

## Abstract

Thiiobenzyhdrazide (Htbh) and its Cu(II) complexes, $\left[\text{Cu}{\left(\text{Htbh}\right)}_{2}{\text{Cl}}_{2}\right]$ and $\left[\text{Cu}{\left(\text{tbh}\right)}_{2}\right]$ were synthesized and characterized by various physicochemical studies. *In vivo* and *in vitro* antitumour activity of Htbh, $\left[\text{Cu}{\left(\text{Htbh}\right)}_{2}{\text{Cl}}_{2}\right]$ and $\left[\text{Cu}{\left(\text{tbh}\right)}_{2}\right]$ has been tested. ${\text{LD}}_{50}$ values were calculated for all the three compounds. It was observed that the antitumour effect of $\left[\text{Cu}{\left(\text{Htbh}\right)}_{2}{\text{Cl}}_{2}\right]$ is maximum. Light microscopic study of the treated tumour mass demonstrated that certain cellular degradation, such as disappearance of mitotic figures, loss in cellular compactness, distortion of nucleus and disruption of cytoplasmic boundaries, takes place in the tumour region of complex treated mice. Further, tumour bearing mice administered with Cu(II) complexes showed reversal of tumour growth associated induction of apoptosis in lymphocytes.


### Antitumour and Immunomodulatory Effects of Cu(II) Complexes of Thiobenzyhdrazide

Nand K. Singh and Saty B. Singh
*Department of Chemistry, Banaras Hindu University,*
*Varanasi-221 005, India A*
Anuraag Shrivastav
*Department of Pathology & Saskatoon Cancer Centre*
*College of Medicine, University of Saskatchewan*
*20 Campus Drive, S7N 4H4, Saskatoon, SK, Canada*

### ABSTRACT

Thiiobenzyhdrazide (Htbh) and its Cu(II) complexes, $[Cu(Htbh)_2Cl_2]$ and $[Cu(tbh)_2]$ were synthesized and characterized by various physicochemical studies. *In vivo* and *in vitro* antitumour activity of Htbh, $[Cu(Htbh)_2Cl_2]$ and $[Cu(tbh)_2]$ has been tested. $LD_{50}$ values were calculated for all the three compounds. It was observed that the antitumour effect of $[Cu(Htbh)_2Cl_2]$ is maximum. Light microscopic study of the treated tumour mass demonstrated that certain cellular degradation, such as disappearance of mitotic figures, loss in cellular compactness, distortion of nucleus and disruption of cytoplasmic boundaries, takes place in the tumour region of complex treated mice. Further, tumour bearing mice administered with Cu(II) complexes showed reversal of tumour growth associated induction of apoptosis in lymphocytes.

### INTRODUCTION

During the past decade after the successful achievement of *cis*-dichlorodiammine platinum(II) (cisplatin), a number of derivatives of thiosemicarbazone, such as 3-ethoxy-2-oxobutyraldehyde *bis*(thiosemicarbazone) copper complexes (Cu-KTS), have been found to exhibit antitumour activity /1,2/ by binding with DNA /3/. In addition, the copper complexes of thiosemicarbazide inactivate lambda phage infectivity and transaction by lambda DNA /4/, and inhibit the activity of RNA dependent DNA polymerase of Rous sarcoma virus /5/. Thiosemicarbazones of 1-formylisoquinoline and 2-formylpyridine and their derivatives were also demonstrated as effective against animal tumour /6,7/ at the molecular level by inhibiting the enzyme ribonucleoside diphosphate reductase and synthesis of DNA /8-10/. Many chemotherapeutic agents have also been reported to possess immunomodulatory properties /11,12/. However, the immunomodulatory potential of the metal complexes with antitumour activity has not been explored adequately. Although thiohydrazides are structurally quite similar to thiosemicarbazides, scarcity of work on the antineoplastic and immunomodulatory activity of transition metal complexes of thiohydrazides prompted us to study the activity of transition metal complexes of thiobenzhydrazide ($C_6H_5C(S)NHNH_2$). In the present investigation we report the synthesis, characterization and antitumour activity of Cu(II) complexes of thiobenzhydrazide (Htbh). Further, we also investigated the immunomodulatory action of these complexes on Dalton's Lymphoma (DL) bearing host.

### MATERIALS AND METHODS

Elemental analyses, magnetic susceptibility, infrared, electronic and ESR spectra of the complexes were recorded as described elsewhere /12/.

### Synthesis of ligand

Carboxymethyldithiobenzoate (2.0g) was dissolved in 1N-NaOH (10 mL, 1 equi) and 10 mL water. The solution was cooled in ice and hydrazine hydrate (0.44g, 2 equi) was added. The thiobenzhydrazide separated immediately and the orange colour of the solution disappeared. A few drops of 1N-HCl were added to the above solution to bring it to pH ~5-6 and the mixture was kept in ice for one hour. The product thus obtained was filtered off, washed with water and dried *in vacuo*. The crude product was recrystallized from hot ethanol (mp 73-75 °C) /13/ (Fig. 1).

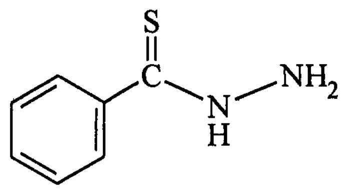

Thiobenzhydrazide
(Hbth)

**Fig. 1:** Thiobenzhydrazide (Hbth)

### Synthesis of Cu(II) complexes

$[Cu(Htbh)_2Cl_2]$ was prepared by adding an ethanol solution of copper(II) chloride, containing a few drops of dil. HCl to the ethanol solution of the thiobenzhydrazide in a 1:2 molar ratio. The complex, which precipitated immediately, was filtered by suction, washed with ethanol and dried *in vacuo*. $[Cu(tbh)_2]$, on the other hand, was prepared by adding an ethanol solution of the ligand (thiobenzhydrazide) to an aqueous solution of copper(II) acetate in a1:2 molar ratio in the presence of sodium acetate (~0.5g) and digesting the reaction mixture for ~30-60 min. The complex thus obtained was filtered by suction, washed successively with water, ethanol and finally with ether, and dried *in vacuo* (Fig. 2).

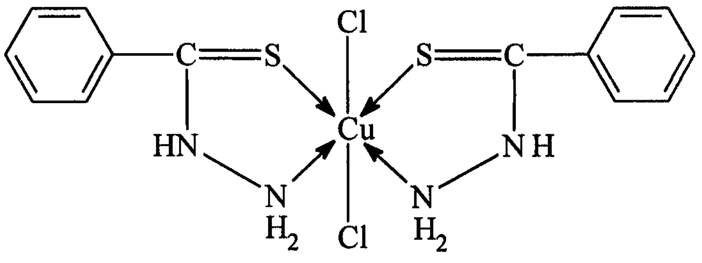

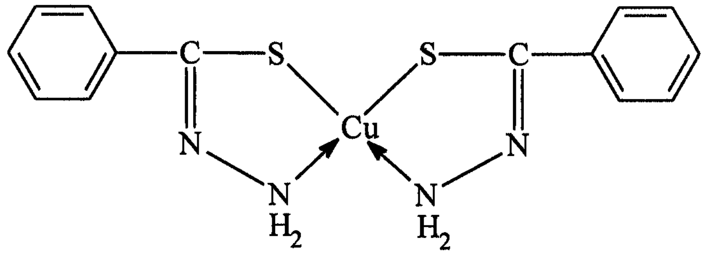

**Fig. 2:** Proposed structure of Cu(II) complexes

### Test for toxicity

DL cells were transplanted in BALB/c mice by an intraperitoneal injection of $5 \times 10^5$ cells/mouse. On day three of transplantation of mammary gland tumour, the test compounds in different doses were injected IP as a single injection. Five animals were used per dose level. Toxic doses were estimated on the basis of survivals on the fifth day of injection (Table I).

**Table I**

Toxic Doses of Htbh and its Cu(II) Complexes

| Complex | HNTD mg/kg body weight | TDH mg/kg body weight | $LD_{50}$ mg/kg body weight | $ID_{50}$ µg/mL |
|---|---|---|---|---|
| Htbh | 68 | 90 | 250 | 5.2 |
| $[Cu(tbh)_2]$ | 50 | 75 | 200 | 2.3 |
| $[Cu(Htbh)_2Cl_2]$ | 30 | 60 | 150 | 1.2 |

HNTD (Highest Non-Toxic Dose): The dose which can be administered without causing hematologic chemical or pathologic abnormalities; doubling this dose causes toxicity.

TDH (Toxic Dose High): Dose which causes abnormalities; doubling this dose produces lethality.

$LD_{50}$ (Lethal Dose 50): Dose that causes death in 50% of animals.

$ID_{50}$ (Inhibition Dose 50): Dose that causes 50% inhibition of tumour growth *in vitro*.

### Dose schedule

Eight to twenty mice were used for each set of experiments. Htbh, $[Cu(tbh)_2]$ and $[Cu(Htbh)_2Cl_2]$ were injected only once IP at appropriate dose levels in mice on day three after tumour transplantation. Compound suspension was freshly prepared in 30% ethanol. Controls were injected with the same volume of 30% ethanol. On alternate days, up to 4 weeks tumour bearing animals were weighed to determine the change in body weight. The acute toxicities of the complexes were determined after 48h of compound injection in tumour bearing mice. $LD_{50}$ values obtained are given in Table I.

### Evaluation of antitumour activity

The therapeutic effectiveness of Htbh, $[Cu(tbh)_2]$ and $[Cu(Htbh)_2Cl_2]$ against DL bearing mice was assessed from the mean survival time of the treated animals (excluding tumour free survivors) divided by mean life span of untreated mice multiplied by 100, giving the T/C percentage. A T/C value of 115 indicates significant activity, wherewas a value for T/C > 125 indicates that the complex is worthy of testing in other tumour systems.

### Histological study

Animals from both control and treated batches were killed at 2-day intervals up to 6 days. Tumour tissue with liver, kidney and spleen was fixed in Bouin's fluid (aqueous), dehydrated, kept in cedar wood soil for 3 days and embedded in paraffin. Paraffin sections of 5 μm were cut, stained in Ehrlich's hematoxylin eosin stain, dehydrated, cleared in xylene, and mounted in DPX. Slides were studied under the light microscope.

All other biological studies were carried out as described elsewhere /12/.

### RESULTS AND DISCUSSION

The molar conductance values of the complexes in DMF demonstrate their non-ionic nature. The magnetic moments of 1.86 – 2.20 B.M. for Cu(II) complexes correspond to the presence of one unpaired electron. The electronic spectrum of $[Cu(Htbh)_2Cl_2]$ shows bands at 10530, 16000 and 25000 $cm^{-1}$, which were assigned to $^2B_{1g} \rightarrow {}^2A_{1g}(\nu_1)$; $^2B_{1g} \rightarrow {}^2B_{2g}(\nu_2)$ and $^2B_{1g} \rightarrow {}^2E_g(\nu_3)$ transitions, respectively. The positions of these bands and their assignment suggest distorted octahedral geometry. A band at 20,410 $cm^{-1}$ in the spectrum of $[Cu(tbh)_2]$ indicates its square planar geometry. The ESR spectra of $[Cu(Htbh)_2Cl_2]$ and $[Cu(tbh)_2]$ in $CHCl_3$ at 77 °K yield a broad signal, from which $g_{iso}$ values have been found to be 2.1221 and 2.0548, respectively. The IR spectrum of the ligand displays bands at 3400, 3220, 1655 and 980 $cm^{-1}$, which may be assigned to ν(NH), $\nu(NH)_2$, $\beta(NH)_2$ and ν(N-N) modes, respectively. The $\nu(NH)_2$ band shows negative shifts of 80 and 60 $cm^{-1}$ in $[Cu(Htbh)_2Cl_2]$ and $[Cu(tbh)_2]$, respectively, whereas the ν(N-N) band shows a positive shif6t of 40 – 45 $cm^{-1}$, indicating that the nitrogen of the amino group is involved in bonding. Thioamide bands I and II undergo positive shifts showing bonding through thione/thiol sulfur.

However, thioamide band-IV [mainly due to ν(C=S)] undergoes a negative shift of 40 $cm^{-1}$ in $[Cu(Htbh)_2Cl_2]$, indicating bonding through 'thione' sulfur. This band is, however, found to be absent in $[Cu(tbh)_2]$ and in place of this a new band appears at 720 $cm^{-1}$, due to ν(C-S), which indicates bonding through 'thiolato' sulfur /13/ (Fig. 2).

### ANTITUMOUR STUDIES

To study the antitumour activity, the ligand and its Cu(II) complexes were administered (60 and 120 mg/kg of mouse weight) to Dalton's Lymphoma (DL) bearing mice. Toxic doses were evaluated in terms of HNTD, TDH and $LD_{50}$ (Table 1). Maximum *in vivo* antitumour activity was found for $[Cu(Htbh)_2Cl_2]$. The enhanced activity of the chloro complex as compared to $[Cu(tbh)_2]$ may be attributed to the presence of labile chlorine, as is observed in the case of cisplatin. Also, the number of DL bearing mice administered with $[Cu(Htbh)_2Cl_2]$ surviving for more than 3 months was found to be maximum (Table 2). We checked the life prolongation effect in DL bearing mice administered with phosphate buffer saline (PBS) alone or containing ligand or its Cu(II) complexes. As shown in Table 2, maximum % T/C was found for $[Cu(Htbh)_2Cl_2]$ followed by $[Cu(tbh)_2]$, whereas the ligand alone failed to increase significantly the life span of tumour bearing mice. Increase in the value of % T/C indicates a prolongation of the life of tumour bearing mice and suggests that such an effect could result either from the direct cytotoxic/cytostatic action of the complexes on tumour cells or from the activation of certain host derived antitumour defense mechanism(s). Therefore, in the next part of the study we carried out investigations to understand the mechanism(s) underlying the prolongation of the life span of tumour bearing mice treated with Cu(II) complexes.

Part of the evidence comes from the experiments in which tumour cells, incubated in the presence or absence of metal complexes, were checked for their effect on viability by MTT assay, in which 3,(4,5-

**Table II**

Screening Data for Htbh, $[Cu(tbh)_2]$ and $[Cu(Htbh)_2Cl_2]$ in the mammary gland tumour bearing mice of $C_3h$ Jax strain

| Dosage in mg/kg weight of mouse | Number of mice injected | | | Post inoculation life span (% T/C) | | | Number of mice surviving more than three months[d] | | |
|---|---|---|---|---|---|---|---|---|---|
| | I | II | III | I | II | III | I | II | III |
| 60 IP | 10/10 | 10/10 | 10/10 | 115 | 178 | 210 | 2(10) | 2(10) | 8(10) |
| 120 IP | 8/8 | 20/20 | 20/20 | 120 | 185 | 218 | 3(8) | 5(20) | 15(20) |

a. Ten injections on alternate days of the following dose was given.
b. Average weight change from day of drug treatment to 30 days.
c. In calculating average survival time, mice surviving 3 months or more were not included.
d. Number in parentheses indicates total number of mice injected.
e. Treatment was initiated on day three after tumour transplantation.

I = Htbh; II = $[Cu(tbh)_2]$; III = $[Cu(Htbh)_2Cl_2]$

dimethylthiazol-2-yl)-2,5-diphenyl tetrazolium bromide) is metabolised to an insoluble coloured formazan salt by mitochondrial enzyme activity of SDH in living cells /14/. As shown in Table 1, treatment of tumour cells with [Cu(Htbh)$_2$Cl$_2$] caused maximum growth inhibition, followed by [Cu(tbh)$_2$] and the ligand, indicating an inhibition of the SDH activity. These results thus indicate a possible decline of the overall metabolic activity of the tumour cells with a concomitant inhibition of the activity of the enzymes involved in halting the process of respiration.

Although the metal complexes showed cytostatic effects on the tumour cells *in vitro*, these results do not necessarily indicate if these cells are actually killed by the direct action of the metal complexes. To check this in the next part of the investigation we studied the effect of the metal complexes on tumour cell killing to

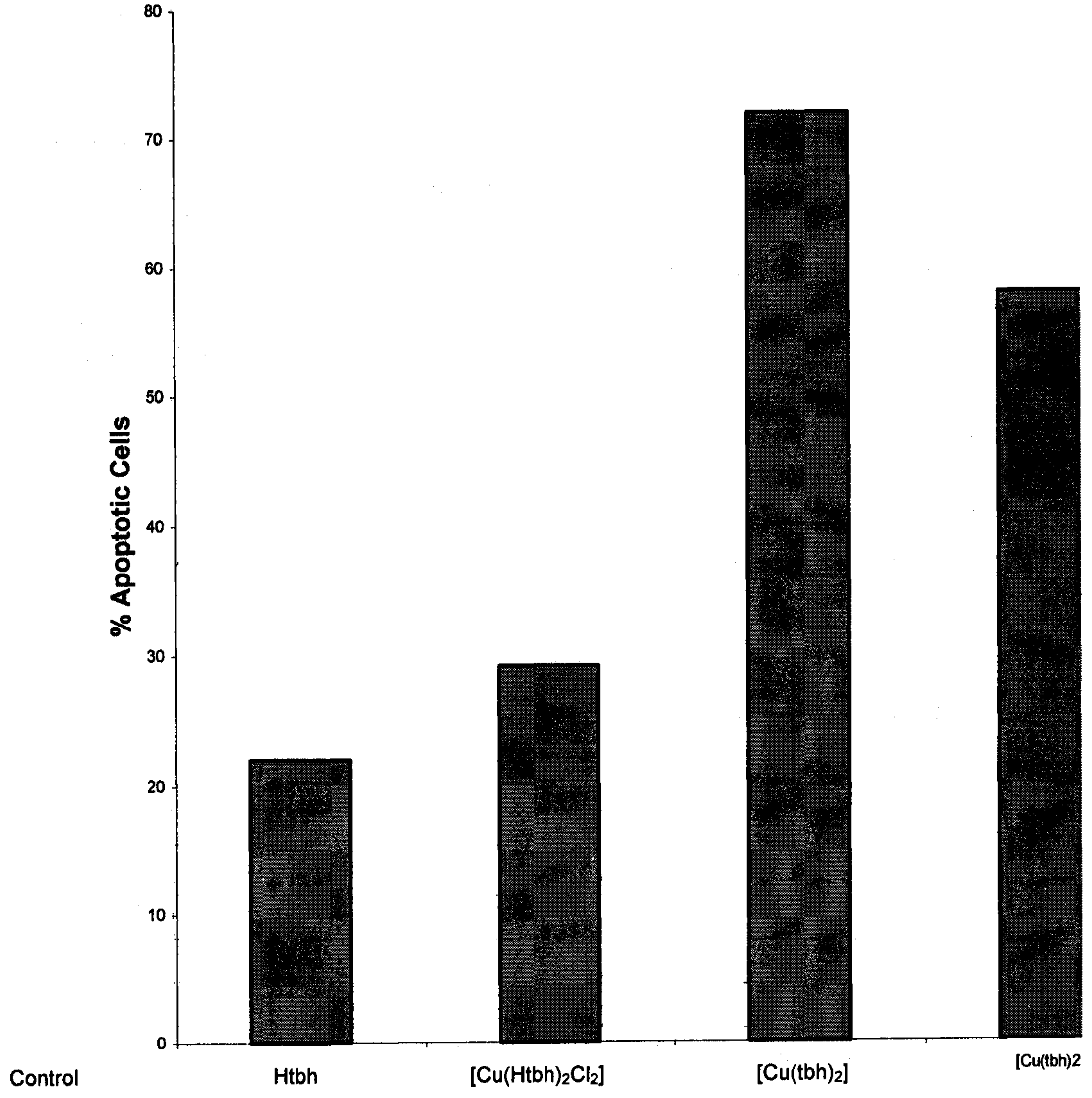

**Fig. 3a:** Effect of Htbh and its Cu(II) complexes on the induction of apoptosis in tumour cells. DL cells were incubated in medium alone or containing Htbh or its Cu(II) complexes (10 μg/mL) for 24 h and the number of cells showing apoptotic morphology was enumerated. Values are the mean of three experiments.

identify the mode of cell death. The results suggest that apoptosis induced in tumour cells treated with [$Cu(Htbh)_2Cl_2$] was found to be most effective in the induction of tumour cell apoptosis (Fig. 3a). The mechanism of the induction of apoptosis remains poorly understood and is thought to be dependent on multiple mechanism(s) ultimately culminating in the activation of DNA cleaving endonucleases /15/. Indeed, results presented in Fig. 3b show that [$Cu(Htbh)_2Cl_2$] and [$Cu(tbh)_2$] cause an increase in the percentage of specific DNA fragmentation, a hallmark feature of apoptosis, indicating that these metal complexes may induce apoptosis culminating in the activation of endonucleases causing DNA fragmentation.

Light microscopic studies reveal that untreated DL cells show compact cellular organization, in which the cells have an oval shaped large nucleus with a single prominent nucleolus. The cytoplasma is well developed and chromatin material is prominent. Cells in the various stages of mitotic division could be seen. A few leucocytes and lymphocytes were observed. The treated animals revealed the loss of cellular compactness of the tumour mass and cytoplasm appears to be distorted with vesicle appearances. The cell walls of most of the cells were ruptured, nuclei became translucent and lost their shapes. The nucleoli material became more

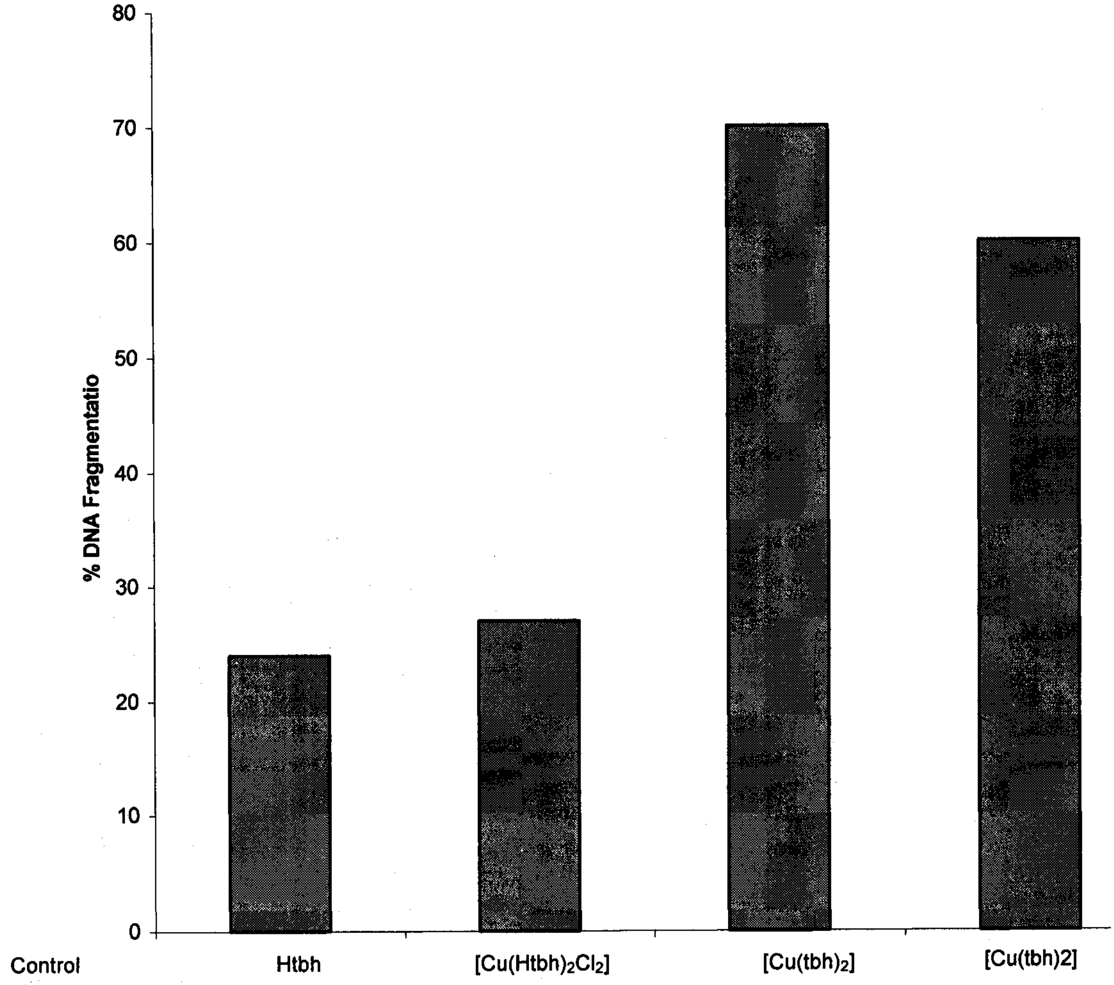

**Fig. 3b:** Effect of Htbh and its Cu(II) complexes on % DNA fragmentation of tumour cells. DL cells were incubated in medium alone or containing Htbh or its Cu(II) complexes (10 μg/mL) for 24 h and the % DNA fragmentation was evaluated. Values are the mean of three experiments.

condensed. The high dose treatment was more effective and cell cytoplasm and the cytoplasmic boundaries between the cells were disrupted. The nucleus became more translucent and nuclear material was in the form of condensed granules. In the case of [Cu(Htbh)$_2$Cl$_2$] a more effective result was obtained, in which the completely destroyed cytoplasmic mass was prominent. A large number of leucocytes, lymphocytes and macrophages appeared. Only a few mitotic figures were observed after ten doses of treatment. The results reported in this study showed that [Cu(Htbh)$_2$Cl$_2$] has a strong antitumour effect and also plays an important role in the tumour regression.

The appearance of lymphocytes and macrophages in the tumour mass after the treatment with metal complexes also suggests that probably the host's immune defence mechanism is increased. This prompted us to investigate if the administration of metal complexes could reverse tumour growth associated induction of apoptosis in various hematopoietic cells. For this, DL bearing mice were administered with Cu(II) complexes, and the percentage of apoptotic thymocyte, splenocyte and bone marrow cells were enumerated. As shown in Fig. 4a, administration of metal complexes in tumour bearing mice resulted in the inhibition of

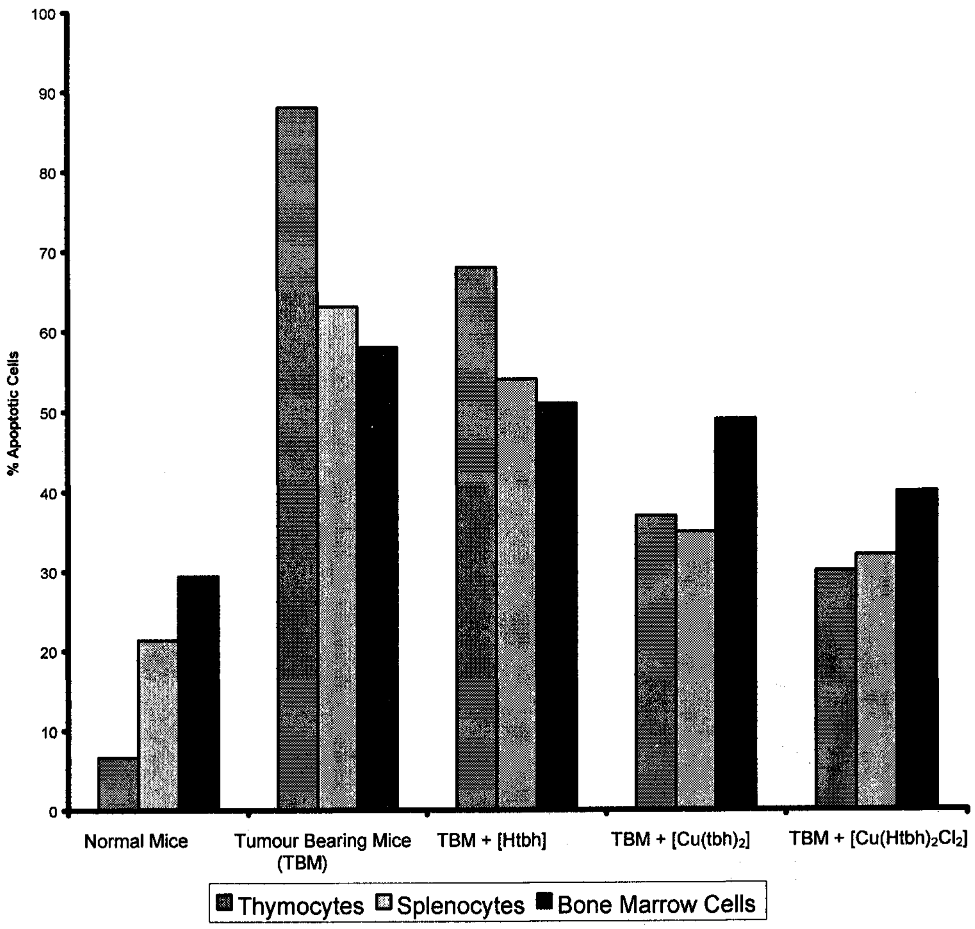

**Fig. 4a:** Effect of *in vivo* administration of Htbh or its Cu(II) complexes on the induction of apoptosis in thymocytes, splenocytes and bone marrow cells of normal or tumour bearing mice treated with ligand or its metal complexes. These cells were enumerated for the number of cells showing apoptotic morphology. Values are the mean of three experiments.

tumour associated apoptosis of thymocyte, splenocyte and bone marrow cells. Similar results were obtained for percentage DNA fragmentation as well (Fig. 4b). The reversal of tumour growth associated inducation of apoptosis of hematopoietic cells by metal complexes is predicted to be due to reduction of tumour load resulting from the cytotoxic effect of metal complexes on tumour cells, leading to a decrease in the tumour associated concentration of apoptotic factors and by the direct protective effect of metal complexes on the hematopoietic cells. Although not very clear, the probability of the latter could be due to the fact that metal complexes can bind to DNA and several proteins in cells, which could result in the protective effect.

The present study shows that the Cu(II) complexes of thiobenzhydrazide are not only active antitumour agents, but are also immunopotentiating agents, on account of their ability to reverse tumour associated immunosuppression. The importance of such work lies in the possibility that the new complexes might be more efficacious drugs for therapeutic use against tumours in view of their dual mechanism of action.

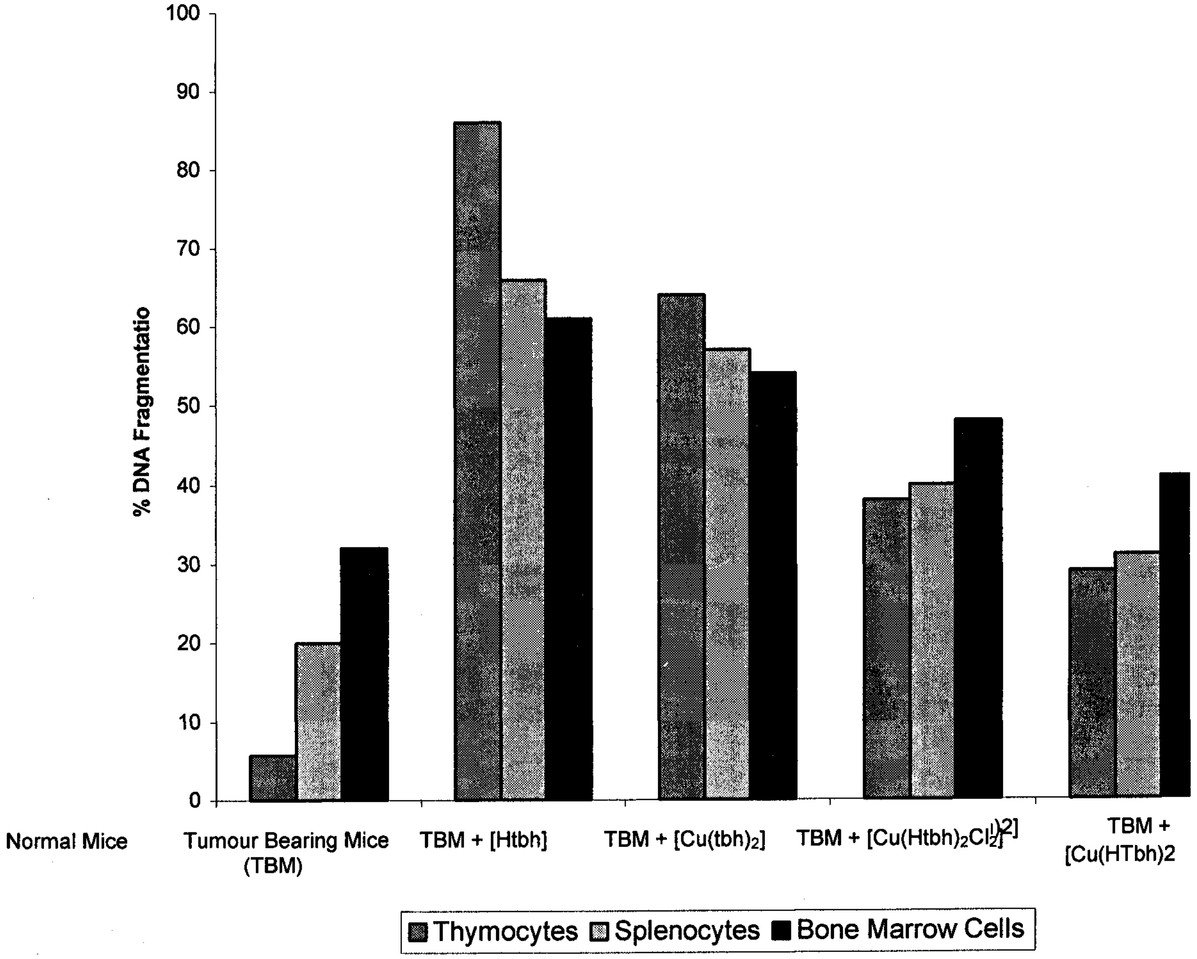

**Fig. 4b:** Effect of *in vivo* administration of Htbh or its Cu(II) complexes on % DNA fragmentation of thymocytes, splenocytes and bone marrow cells of normal or tumour bearing mice treated with ligand or its metal complexes. These cells were checked for % DNA fragmentation. Values are the mean of three experiments.

### ACKNOWLEDGEMENT

The authors thank Prof. G.C. Prasad, Department of Shalya Shalakya, Institute of Medical Sciences, and the Coordinator, School of Biotechnology, Banaras Hindu University, Varnasi, India for their help in evaluating the biological data.

### REFERENCES

1. H. Petering, H. Buskirk and J. Crim, *Cancer Res.*, **27**, 1115 (1967).
2. J. Crim and H. Petering, *Cancer Res.*, **27**, 1278 (1967).
3. P. Mikelens, B. Woodsen and W. Levinson, *Biochem. Pharm.*, **24**, 821 (1976).
4. W. Levinson and R. Helling, *Antimicrobial Agents and Chemotherapy*, **9**, 160 (1976).
5. W.C. Kaska, C. Carrano, J. Michalowski, J. Jackson and W. Levinson, *Bioinorg. Chem.*, **8**, 225 (1978).
6. E.J. Blanz, F.A. French, D. Ameual and D.A. French, *J. Med. Chem.*, **13**, 1124 (1970).
7. F.A. French, E.J. Blanz, S.C. Shaddix and R.N. Brockman, *J. Med. Chem.*, **17**, 172 (1974).
8. A. Sartorelli, *Biophys. Res. Commun.*, **27**, 26 (1967).
9. A. Sartorelli, *Cancer Res.*, **29**, 2292 (1969).
10. E.C. Moore, B.A. Booth and A.C. Santorelli, *Cancer Res.*, **31**, 235 (1971).
11. A.K. Lichtenstein and D. Pende, *Cancer Res.*, **46**, 639 (1986).
12. A. Shrivastav, N.K. Singh and S.M. Singh, *Bioorg. & Med. Chem.*, **10**, 887 (2002).
13. N.K. Singh and U. Sharma, *Synth. React. Inorg. Met. Org. Chem.*, **19**, 235 (1989).
14. T.M. Buttke and P.A. Sandstrom, *Immunol. Today*, **15**, 7 (1994).
15. P. Ranjan, A. Sodhi and S.M. Singh, *Anticancer Drugs*, **9**, 333 (1998).

